# The Hospital

**Published:** 1917-11-24

**Authors:** 


THE
### INSTITUTIONAL WORKER
**Being a Special Supplement to "The Hospital"**

### OUR BUREAU OF INFORMATION.

### Rules for Correspondents.

1. Every letter must be accompanied by the coupon to be cut from the back cover (inside page) of The Hospital, current issue, and must contain the name and address of the correspondent with pseudonym for publication if desired. Replies by post cannot be given save under exceptional circumstances at the Editor's discretion.

2. Letters from Approved Homes in reply to special needs published in the Bureau should state terms and full particulars, and be sent prepaid under cover to the Editor of the Bureau with name written across coupon for identification.

3. Proprietors of Homes which have not yet been entered on the List of Approved Homes, but have spare accommodation likely to suit special needs, are invited to write for an application form for registration. The fee for registration, which includes two announcements of the Home in the Bureau and other privileges, is 10s.

4. All communications to be addressed to the Editor of The Hospital, 28 Southampton Street, Strand, London, W.C. 2, and marked "Bureau of Information."

### An Isolation Hospital Difficulty.

### Answer to October Question.

**By "Housekeeper."**

The question was:

**In an isolation hospital wards are often closed for months at a time. In spite of good ventilation and dry weather, the enamel of the bedsteads peels off, and the stoves get rusty very quickly. Explain fully what can be done to prevent this.**

The question of keeping all ironwork in order is a very difficult one. If a building is closed entirely, with no one in residence, it is almost impossible, as such things as bedsteads and fireplaces need daily care, especially in damp or foggy weather, the best rust preventive being a good fire occasionally. Where a caretaker is in charge of a building it is best to arrange with him that when the weather is at all damp he shall each day go over the metal of the bedsteads with a rag that has been saturated with pure mutton fat. In fine weather this should be done once a week at least. If, when fires are done with, all grates and fireplaces are given a coat of liquid Brunswick black, and blacklead applied and polished in the usual way, this should keep them in order when the hospital is in use.

When the building has to be left with no one to look after these things all bedsteads should be very carefully examined for scratches in the varnish of the ironwork. It is at such spots that the peeling takes place, for the iron is thus exposed to the action of the atmosphere. If the varnish is in good condition, with just a slight scratch here and there, such scratches should be covered with a clear varnish; but if the scratches are numerous and the varnish chipped, the only thing to be done is to varnish the bedstead all over, leaving it to dry for twenty-four hours at least, and longer if possible. When the varnish is thoroughly dry, warm some vaseline (the rough kind used by engineers will do quite well) and mix enough powdered blacklead with it to make a fairly soft paste. This mixture should be applied to all the varnished metal of the bedstead, making sure that every bit of metal is covered; it is easier to apply with a bit of old flannel than with either a brush or a cotton rag. For the wire springs of the bedsteads a rag saturated with mutton fat is best, but the wire must have a very thorough rubbing on both sides of the springs.

After the grates and fireplaces have been treated with a coat of Brunswick black a few pieces of ordinary red brick placed in the grate will absorb a lot of the damp and prevent it settling on the ironwork. A shallow box of lime placed on the hearth is also good for this purpose, but the lime requires changing from time to time. Care should be taken to see that the lime so used is slaked lime, as unslaked lime generates a great heat when it becomes damp, and this has been known to cause fires. Slaked lime is, however, perfectly safe.

### What to Write in a Visitors' Book.

**By Assistant Matron.**

In a paragraph in The Hospital of October 13 it is suggested that the writing of a report in the visitors' book requires a trained pen as well as a trained eye. This doubtless is so, but if a house visitors' book, on each page of which were printed the following questions, were kept in a convenient place in the institution, the writing of an accurate and intelligent report would be greatly simplified.

### Specimen Questions in the Visitors' Book.

1. Did you find every part of the premises clean and in good order, and did the sanitary arrangements appear satisfactory? (Visitors should particularly observe whether all baths, lavatories, sinks, etc., are in good order, and that the heating and ventilation are properly attended to.)

2. Did you observe that any part either of the buildings or furniture required repairing?

3. Were the beds and bedding, appliances and utensils perfectly clean?

4. Were the bread, meat, vegetables, milk, and other provisions supplied to the patients wholesome and well cooked? Dinner is served at one o'clock. Tea is served at four o'clock.

5. Did you ascertain from the patients that they were satisfied with the attendance of the medical and other officers and servants belonging to the institution? (These inquiries should be made when the patients are by themselves and under no restraint.)

6. Did you see, as far as possible, if every person appeared to be regularly discharging his or her duties in accordance with the rules and regulations of the institution?

General remarks ....................

(Signed) ............ House Visitor.

### The Scheme in Practice.

In one small provincial hospital where such a book is used, the house visitors, who are appointed periodically by the committee, pay frequent and unannounced visits at any hour of the day, and visit the wards and all parts of the building unaccompanied by any officer of the institution. They therefore have every opportunity of individual observation, and are able to elicit any information they require from the patients, who, of course, talk more freely under such circumstances than if the visitor were accompanied by an officer. On leaving, the visitor makes his report, and this is examined at each meeting of the committee, thus enabling them to determine whether the institution is conducted in a satisfactory manner or otherwise.

### Questions for November.

**1. The Equipment of an Out-Patient Department.**

State what you consider necessary for the general equipment of a new Out-Patient Department and Accident Room, and the procedure to be adopted for the provision of such requirements.

**2. How to Organise a Mothers' and Babies' Welcome.**

Outline a simple scheme which a midwife working independently and single-handed in a scattered country district could adopt to start a "Mothers' and Babies' Welcome."

RULES.

The following rules must be observed:—
1. Contributions must be written on one side of the paper. Brevity and terseness are desirable features. The MSS. must bear the name and address of the sender and be accompanied by coupon to be cut from the back cover (inside page) of the current issue of THE HOSPITAL. A pseudonym must be chosen if the name is not to be published.
2. Contributions must be addressed to the Editor of THE HOSPITAL, Institutional Worker Supplement, 28 & 29 Southampton Street, Strand, London, W.C. 2, should reach him before the end of the current month, and be marked in the left-hand corner "Facts and Figures."

**A minimum payment of Half-a-Guinea will be made for each published answer.**

### QUESTIONS INVITED.

IN connection with our Question Box, we offer every month five shillings each for the two best questions which are sent in for consideration. The questions may be on any institutional subject and concern any department, from the secretary's to the porter's, or the matron's to the domestic staff; the one essential is that they must be practical and deal with points that have a definite relation to hospital or institutional administration, upkeep, finance, or management. Points relating to artificers' work, housekeeping, the laundry, out-patients, and other practical matters will be welcome. It is hoped that thus, when difficult or doubtful points arise in the course of their work, institutional workers will be encouraged to put them in the form of a question and send them to the Editor, THE HOSPITAL Bureau of Information, 28 Southampton Street, Strand, W.C. 2, marked "Question Box."

The best questions will be published in due course and our readers will be given the opportunity of answering them. Thus all institutional workers may be able to co-operate with the view of helping the work and smoothing the difficulties of each other. In short, we want the Question Box of the *Institutional Worker* to be freely used by every worker habitually.

### ENQUIRIES AND ANSWERS.

### *THE SICK AND IN NEED.*

**Case of Hip Disease.**

WE suggest that you apply to the Royal Sea-Bathing Hospital, Margate, on behalf of the girl suffering from hip disease. Write to the London Office, 13 Charing Cross Road, London, S.W. Patients are admitted to this hospital with a governor's letter and a payment of from £1 12s. to £2 8s. for four weeks.—H. M. R.

### *EMPLOYMENT AND TRAINING.*

**Period of Training for Dispenser's Assistant.**

THE period of training required for the assistant dispenser's certificate granted by the Society of Apothecaries, which is the lowest qualification, is about six months. The coaching charges range from £8 8s., and the examination fee is £5 5s. You can obtain a syllabus and form of regulations by applying to the Secretary, Apothecaries' Hall, Blackfriars, London, E.C. You might possibly make arrangements with a local chemist, or a few hospitals receive pupil dispensers, such as the Women's Hospital, Birmingham.—P. D. A.

**Medico-Psychological Certificate.**

YOUR best course would be to apply to the medical superintendent of one of the county asylums, or to the Clerk of the Metropolitan Asylums Board, Victoria Embankment, London, W.C. Candidates for the certificate of the Medico-Psychological Association must have completed a three years' course of training at a recognised asylum for the insane; most asylums in the United Kingdom of 100 beds and upwards are recognised by the Association.—OLGA.

**The Way to Succeed in Infant Welfare Work.**

DR. HARRY SHORE, the medical officer of health for Walsall, gives some hints for making the movement for infant welfare a success. No effort should be spared to attract the ideal or desirable cases. Every consideration should be shown by the staff, and care taken to avoid any semblance of parish medical relief. It is well to lay stress on the fact that if a case is deemed suitable, attendance at the centre is a "Burgess Right." The staff should study the local peculiarities of the working classes and endeavour to adopt methods and manners to harmonise with and conciliate them. To make any lasting impression, and to be able to lead—for the working classes cannot be driven—the staff must establish an atmosphere of freedom from restraint of confidence, or, to quote an oft-expressed phrase, "Be a person that they feel they can talk to." The problem of each patient should be studied individually. Unless this is done, the work may easily become mechanical, even though scientifically done. However able or extensive the efforts of a centre may be, the question of environments and the ingrained habits of parents play important parts; in fact, may definitely limit the extent to which progress may be made.

### *MISCELLANEOUS.*

**Hospital Appeals.**

As the struggle to make ends meet becomes increasingly greater, so must the hospital treasurer make a corresponding increase in his efforts to bring his needs home to the public. Failure to obtain the requisite support is, as a rule, an indication only of failure to make the needs known. Some whose attention is not attracted by the work of a hospital as a whole, may have their interest stimulated in the work by some special department. Hospital supplies and general requirements are provided by a thousand-and-one different trades and professions. The possibility of attracting interest in the hospital through mutual interests of this character—through the common brotherhood of handicrafts—is limitless. In these days, too, how many merchants would not be glad to purchase at bottom prices and without commission the numerous domestic and other supplies required?—OPTIMIST.

### *WAR MATTERS.*

**Supplies for Auxiliary Hospitals.**

AUXILIARY hospitals in receipt of maintenance allowance for soldiers are not allowed by the War Office the privilege of purchasing supplies through the War Department; the fact that the allowance, which averages about 4s. per day, is insufficient to meet the expenditure incurred, does not entitle auxiliary hospitals to any privileges of this character. There is no doubt that if the restriction were removed, the hospitals receiving soldiers could reduce considerably the sum expended on provisions.—STEWARD.

### *THE HOUSEKEEPERS' DEPARTMENT.*

**Economy in Fuel and Light in Nurses' Home.**

THE amount of coal used in the nurses' home and the amount of electricity or gas should be kept distinct from that used in the hospital. Of course, where there is a separate building for the nurses' home, with its own plant for lighting and heating, there is no difficulty in ascertaining exactly what these items cost. Meters for measuring the amount of gas or electricity should be placed on each floor in the home, and nurses should be informed when any extra amount is registered. At the London Hospital a meter is attached to each bathroom, and thus any waste of hot baths is easily detected.—HOME SISTER.

